# Conditioned Media from Human Adipose Tissue-Derived Mesenchymal Stem Cells and Umbilical Cord-Derived Mesenchymal Stem Cells Efficiently Induced the Apoptosis and Differentiation in Human Glioma Cell Lines In Vitro 

**DOI:** 10.1155/2014/109389

**Published:** 2014-05-27

**Authors:** Chao Yang, Deqiang Lei, Weixiang Ouyang, Jinghua Ren, Huiyu Li, Jingqiong Hu, Shiang Huang

**Affiliations:** ^1^Stem Cell Center, Wuhan Union Hospital, Tongji Medical College, Huazhong University of Science and Technology, Wuhan, Hubei 430022, China; ^2^Neurosurgery Department, Wuhan Union Hospital, Tongji Medical College, Huazhong University of Science and Technology, Wuhan, Hubei 430022, China; ^3^Department of Gynaecology and Obstetrics, Wuhan Union Hospital, Tongji Medical College, Huazhong University of Science and Technology, Wuhan, Hubei 430022, China; ^4^Cancer Center, Wuhan Union Hospital, Tongji Medical College, Huazhong University of Science and Technology, Wuhan, Hubei 430022, China

## Abstract

Human mesenchymal stem cells (MSCs) have an intrinsic property for homing towards tumor sites and can be used as tumor-tropic vectors for tumor therapy. But very limited studies investigated the antitumor properties of MSCs themselves. In this study we investigated the antiglioma properties of two easily accessible MSCs, namely, human adipose tissue-derived mesenchymal stem cells (ASCs) and umbilical cord-derived mesenchymal stem cells (UC-MSCs). We found (1) MSC conditioned media can significantly inhibit the growth of human U251 glioma cell line; (2) MSC conditioned media can significantly induce apoptosis in human U251 cell line; (3) real-time PCR experiments showed significant upregulation of apoptotic genes of both caspase-3 and caspase-9 and significant downregulation of antiapoptotic genes such as survivin and XIAP after MSC conditioned media induction in U 251 cells; (4) furthermore, MSCs conditioned media culture induced rapid and complete differentiation in U251 cells. These results indicate MSCs can efficiently induce both apoptosis and differentiation in U251 human glioma cell line. Whereas UC-MSCs are more efficient for apoptosis induction than ASCs, their capability of differentiation induction is not distinguishable from each other. Our findings suggest MSCs themselves have favorable antitumor characteristics and should be further explored in future glioma therapy.

## 1. Introduction

Gliomas are the most common malignant cancer affecting the central nervous system, with a very poor prognosis, in particular with high-grade tumors such as glioblastoma multiforme [1–3]. Current therapy includes surgery, radiation, and chemotherapy. But these treatments are rarely curative. Gliomas usually grow in a highly invasive manner and always infiltrate neighboring tissues and therefore surgical resection rarely removes the tumor completely. After a certain period, gliomas inevitably recur and finally cause the death of the patient. Radiation therapy usually irradiates normal brain tissues surrounding the tumor as well, therefore causing multiple side effects. Chemotherapy is also with limited effects, since most chemicals have difficulty in crossing the blood brain barrier [4]. Temozolomide is the most effective chemotherapeutic reagent but can usually prolong the lifespan for only 3–6 months in some patients [5, 6], with side effects. Novel therapy approaches include combinations of molecular targeted agents such as epidermal growth factor receptor, vascular endothelial growth factor receptor, and mammalian target of rapamycin. But these again have only very limited effects [7]. Overall, gliomas have a poor prognosis and developing novel modalities to increase antiglioma effects and decrease side effects is necessary.

Mesenchymal stem cells (MSCs) are a population of stem cells with self-renewal and multipotentiality which hold great promise for regenerative medicine [8, 9]. In recent years, MSCs have gained increased attention because it has been shown that these cells have an intrinsic property for homing towards tumor sites and can be used as tumor-tropic vectors for tumor therapy [10–17]. Tumor cell-derived substances and factors associated with tumor-induced inflammation and tumor neovascularization can specifically attract stem cells to invasive gliomas. Injected mesenchymal stem cells engineered to produce antitumor substances have shown strong therapeutic effects in experimental glioma models. But very limited studies investigated the antitumor properties of MSCs themselves. There are some preliminary experiments showing that MSCs can suppress human glioma growth [18], but the exact mechanisms remain poorly studied.

To further explore this phenomenon, we examined the interaction of two different types of mesenchymal stem cells, namely, human adipose tissue-derived mesenchymal stem cells (ASCs) and umbilical cord-derived mesenchymal stem cells (UC-MSCs), with the U251 human glioma cell line. Both types of MSCs are easily accessible and have comparative characteristics. We found that conditioned media from both types of MSCs can significantly induce apoptosis in U251 cells. Interestingly, we found a strong effect of MSC conditioned medium on induction of differentiation of U251 glioma cells. Induced U251 cells are evidenced by a morphology similar to normal glial cells and dramatically decreased tumor infiltration ability. These results indicate human mesenchymal stem cells can efficiently induce both apoptosis and differentiation in the U251 human glioma cell line. Our findings suggest that MSCs themselves have favorable antitumor characteristics and should be further explored in future glioma therapy.

## 2. Material and Methods

### 2.1. Isolation, Culture, and Phenotyping of ASCs and UC-MSCs

Human subcutaneous adipose tissues and umbilical cords were obtained from mothers (18–30 years old) planning on cesarean sections after obtaining written informed consent and approval by the Ethics Committee of Wuhan Union Hospital. ASCs and UC-MSCs were isolated and amplified as described elsewhere [19]. ASCs and UC-MSCs were analyzed by multichannel flow cytometry using a standard Becton-Dickinson FACS Aria instrument and the CellQuest Pro software (BD Biosciences).

### 2.2. Multidifferentiation Capabilities of ASCs and UC-MSCs

Adipogenic, osteogenic, and neurogenic differentiation experiments were performed as described elsewhere [19]. Briefly, for adipogenic differentiation, ASCs and UC-MSCs were cultured in DMEM/F12 supplemented with 10% FBS, 1 mM dexamethasone, 0.5 mM methylisobutylxanthine, 10 mg/mL insulin, and 100 mM indomethacin (all from Sigma) for 3 weeks and analyzed by Oil Red O (Sigma) staining.

For osteogenic differentiation, ASCs and UC-MSCs were cultured in DMEM/F12 supplemented with 10% FBS, 0.1 mM dexamethasone, 10 mM b-glycerophosphate, and 50 mM ascorbic acid (all from Sigma) for about 3 weeks and assayed by alizarin red (Sigma) staining.

For neurogenic differentiation, ASCs and UC-MSCs were cultured in DMEM/F12 supplemented with 5 *μ*mol/L retinoic acids (RA) for 6–10 days and analyzed by immunofluorescence microscopy [19].

### 2.3. U251 Cell Line Cultivation

The U251 cell line was purchased from the cell bank of the Chinese Academy of Sciences, Shanghai, China. The cells were maintained in DMEM/F12 supplemented with 10% fetal bovine serum (FBS) (Hyclone, Logan, UT) and 1% penicillin-streptomycin (Invitrogen, Carlsbad, CA).

### 2.4. Harvest of ASC-Conditioned Medium and UC-MSC-Conditioned Medium

ASCs and UC-MSCs at passage 3 were cultured in mesenchymal stem cell growth medium (which is comprised of DMEM/F12 supplemented with 10% fetal bovine serum) until cells were approximately 80% confluent; at that time the medium was replaced with serum-free DMEM/F12. Following incubation in serum-free medium for 48 h, the conditioned medium was collected as* ASC-conditioned medium* (ASC-CM)* and UC-MSC-conditioned medium (UC-MSC-CM)*. The conditioned medium was centrifuged at 1000 rpm for 5 min, filtered through a 0.22 *μ*m syringe filter, and conserved at 4°C until use.

### 2.5. CCK-8 Cell Proliferation Assay

U251 cells were trypsinized and adjusted to a concentration of 2 × 10^4^/mL, and then 100 *μ*L of cell suspension (5,000 cells per well) was seeded in 96-well plates (Costar). 6 hours later, the medium was replaced with either* ASC-conditioned medium or UC-MSC-conditioned medium*. 24 hours later, CCK-8 (10 *μ*L/well) was added to experimental wells and control wells (U251 cells cultured alone), and after 4 hours of incubation, their absorbance values at 450 nm were measured by enzyme immunoassay analyzer, and the mean values were calculated. The experiment was performed three times (*n* = 3) with 3 wells per condition each time.

### 2.6. Flow Cytometric Assay for Apoptosis Using Annexin V and Propidium Iodide

U251 cells were plated in 6-well plates and grown until confluency. The medium was replaced with serum-free ASC-conditioned medium or UC-MSC-conditioned medium and the induction was carried out for two consecutive days. The experiment was performed three times (*n* = 3) with 3 wells per condition each time. 48 hours after induction, U251 cells were digested with 0.2% trypsin for 10 minutes at 37°C. Dissociated cells were washed with phosphate-buffered saline (PBS) two times, resuspended in 500 *μ*L binding buffer, and then incubated for 1 hour at room temperature in the presence of 0.5 *μ*g/mL annexin V-FITC (R&D) and 5 *μ*L propidium iodide for 5 minutes in bindingbuffer as described by the manufacturer. After incubation, the cells were analyzed by flow cytometry.

### 2.7. Real-Time PCR Assay for Apoptotic Induction of U251

RNA was isolated from U251 human glioma cells before and after the induction using Trizol reagent (Invitrogen, Carlsbad, CD, USA). cDNA was transcribed using superscript III first strand cDNA synthesis kit following the manufacturer's instructions (Invitrogen, Carlsbad, CD, USA). Quantitative real-time PCR was performed with SYBR Green PCR reagents on an ABI Prism 7300 detection system (Applied Biosystems, Foster City, CA). Beta-actin was used as an internal control. The normalized fold expression was obtained using the 2^−ΔΔCT^ method. Primers used for real-time PCR are summarized in Table 1.

### 2.8. Immunostaining and Cellomics High Content Screening (HCS) System Assay for Glial Cell Differentiation

U251 cells were plated in 6-well plates and grown until confluency. The medium was replaced with serum-free ASC-conditioned medium and UC-MSC-conditioned medium and the induction was carried out for two consecutive days. U251 cells were then fixed with 4% paraformaldehyde for 15 min, followed by three washes with phosphate-buffered saline (PBS). After a 1-hour blocking with 0.2% Triton X-100 and 3% goat serum in PBS, the cells were incubated with primary antibodies overnight at 4°C. The primary antibodies used in this study include rabbit polyclonal GFAP antibody (1 : 200 dilution; Santa cruz) and mouse monoclonal Tuj1 (1 : 200 dilution; Millipore); subsequently, the cells were washed with PBS three times and then incubated with Dylight 549 goat anti-rabbit secondary antibody (Jackson Immunoresearch Laboratories) and Dylight 488 goat anti-mouse secondary antibody (Jackson) and finally stained with 4′,6′-diamidino-2-phenylindole hydrochloride (DAPI) for 5 minutes.

For quantitative imaging, fluorescent images of the cells were collected automatically by use of the Cellomics high content screening (HCS) system using a 10x objective lens. For axon outgrowth assays, images of GFAP and DAPI staining were analyzed using Neuronal Profiling BioApplication software (Cellomics).

### 2.9. Tumor Cell Migration and Infiltration Assay

For tumor cell migration assay, U251 cells and U87 cells were plated in 6-well plates and grown until confluency. A scratch was made in the confluent monolayer by using a 200 *μ*L pipet tip. The medium was replaced with serum-free ASC-conditioned medium and UC-MSC-conditioned medium and the induction was carried out for two consecutive days.

For the Matrigel tumor cell invasion assay, 2.5 × 10^4^ U251 cells or 10^4^ U87 cells (normally cultured or induced in serum-free ASC-conditioned medium/UC-MSC-conditioned medium for two days) were placed in transwells with 8 *μ*m pore size polycarbonate filters (Corning Incorporated, Corning, NY), precoated with 30 uL of 1 : 6 diluted Matrigel (BD Biosciences). The lower wells were filled with 500 *μ*L of DMEM with 10% fetal bovine serum. The cells were incubated at 37°C for 24 hours. Nonmigratory cells on the upper surface of the transwells were removed with a cotton swab, and the migratory cells from the lower surface of the transwell were fixed in 70% methanol and stained with 1% crystal violet. Cells were photographed and cell numbers in four different fields were counted under 100x magnification for each condition. Average cells per field were calculated. Experiments were done in triplicates on three times independently.

### 2.10. Preparation of ASC-CM and UC-MSC-CM and Protein Microarray Analysis of U251 Cells for Apoptosis Related Proteins

ASCs and UC-MSCs at passage 3 were cultured in mesenchymal stem cell growth medium (which is comprised of DMEM/F12 supplemented with 10% fetal bovine serum) until cells were approximately 80% confluent; the medium was replaced with serum-free DMEM/F12. Two days later, the conditioned medium was harvested as ASC-CM and UC-MSC-CM. U251 cells were cultured in DMEM/F12 supplemented with 10% fetal bovine serum (FBS) (Hyclone, Logan, UT) and 1% penicillin-streptomycin (Invitrogen, Carlsbad, CA) until confluency. The medium was replaced with DMEM/F12 for two days. Then U251 cells were induced using either* ASC-CM or UC-MSC-CM. 48 hours *following induction, U251 cells were collected and lysed using RayBio lysis buffer. The cell lysates were assayed by RayBio AAH-APO-1 (RayBiotech, Inc., Norcross, GA), which can simultaneously detect the expression levels of 43 human apoptotic related proteins in cell lysates.

### 2.11. Statistical Analysis

All values are expressed as mean ± SD. Comparisons between two groups were analyzed by Student's *t*-test, and comparisons between more than two groups were analyzed by ANOVA. A value of *P* < 0.05 was considered statistically significant. All analyses were performed with SPSS 16.0.

## 3. Results

### 3.1. Morphologies of ASCs and UC-MSCs

ASCs morphology and UC-MSCs morphology are basically indistinguishable from each other. Both displayed spindle or whirlpool-like morphology. Primary ASCs and UC-MSCs usually adhered within 24–48 hours after plating. These cells proliferated rapidly within 5–7 days and gradually fused into a single layer (see Supplementary Figure 1 in Supplementary Material available online at http://dx.doi.org/10.1155/2014/109389) and have a very uniform fibroblast-like morphology by passage 3.

### 3.2. Flow Cytometry

Multichannel flow cytometric assays for surface receptor molecule expression were performed. ASCs and UC-MSCs both are usually positive for CD13, CD44, CD90, and CD105 expression and negative for CD14, CD45, and CD34 expression. Representative flow cytometry results are shown in Supplementary Figure 2. Statistical analysis showed the phenotypes of ASCs and UC-MSCs are basically indistinguishable from each other (data not shown).

### 3.3. Multidifferentiation Capabilities of ASCs and UC-MSCs

ASCs and UC-MSCs were both able to efficiently differentiate into adipocytes, osteocytes, and neurons and representative pictures are shown in Supplementary Figure 3.

### 3.4. Both ASC-Conditioned Medium and UC-MSC-Conditioned Medium Significantly Inhibited the Proliferation of U251 Cells

**Figure 1:**
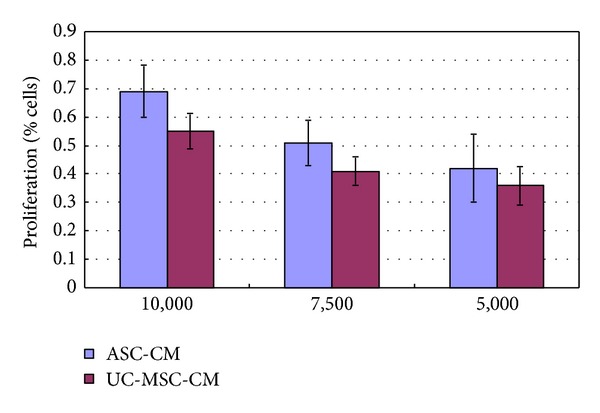
Growth inhibition of U251 glioma cells in MSCs conditioned media. The proliferation of U251 cells was inhibited by conditioned media from both ASC-CM and UC-MSC-CM. The number of cells in control cultures of U251 glioma cells was taken as 100%. The effect on proliferation of U251 under experimental conditions was calculated in relation to single cultures of U251 cells. Numbers on the *X*-axis indicated plated cell number per well.

In order to compare the growth curves of U251 in different conditioned medium, CCK-8 assay was performed. As is shown in Figure 1, both ASC-conditioned medium and UC-MSC-conditioned medium significantly inhibited the proliferation of U251 cells. The growth inhibition effect weakened with increased cell number per well. More significant growth inhibition is seen in UC-MSCs treated cultures, with inhibition being more than 50%.

**Figure 2:**
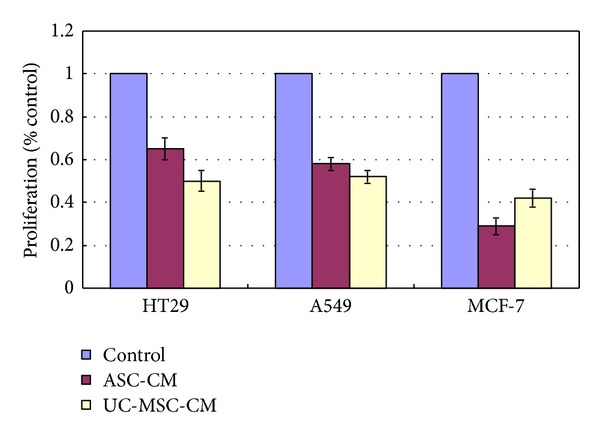
Growth inhibition of HT 29, A549, and MCF-7 cell lines in MSCs conditioned media.

Interestingly, we also found that the growth of several other tumor cell lines, including lung cancer cell line A549, rectal cancer cell line HT29, and breast cancer cell line MCF-7, was inhibited by MSC conditioned media (Figure 2).

### 3.5. Apoptosis Assay Using Both Flow Cytometry and Quantitative Real-Time PCR Confirmed Increased Apoptosis of U251 Human Glioma Cells in MSC Conditioned Medium Culture

**Figure 3:**
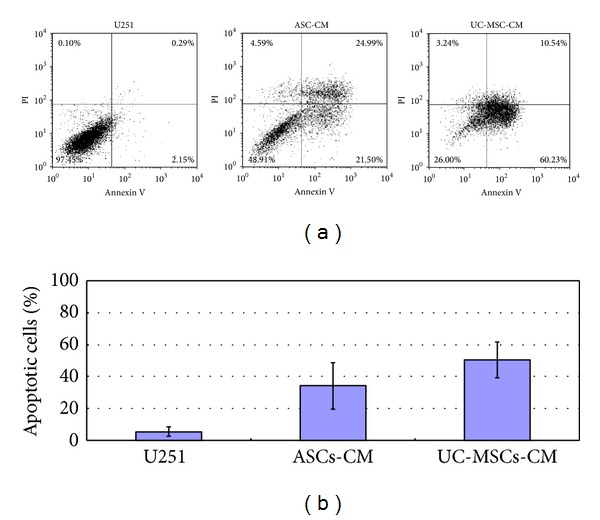
Annexin and PI flow cytometric assay. (a) Representative annexin and PI flow cytometric picture of U251 cells under ASC- and UC-MSC-conditioned media coculture conditions. (b) Statistical results of three independent flow experiments.

In order to find out whether the significant inhibition of growth in U251 cells is due to growth retardation or elevated apoptosis, we did apoptosis assay using both flow cytometry and quantitative real-time PCR. Annexin and PI flow cytometric assays showed that UC-MSCs conditioned medium induced more prominent apoptosis in U251 cells than ASCs conditioned medium: 50.52 ± 11.23% apoptosis in U251 cells for UC-MSCs conditioned medium culture and 34.28 ± 14.45% apoptosis in U251 cells for ASCs conditioned medium culture, as is shown in Figure 3.

Caspases play a key role during the execution phase in various forms of apoptosis. Survivin and XIAP are identified in U251 cells and were assumed to act as antiapoptosis factors. To explore the mechanism of MSC-induced cell death of glioma cells, we did quantitative real-time PCR for caspases 3 and 9, survivin, and XIAP.

Quantitative real-time PCR showed much more increased caspase 3 and caspase 9 expression and much lower survivin and XIAP expression after conditioned medium treatment in U251 cells. After addition of MSCs conditioned medium,* survivin* RNA expression decreased to only 20% of the level of that before treatment, whereas the increase of caspase 9 and caspase 3 expressions is around 5-fold to 10-fold, respectively.

**Figure 4:**
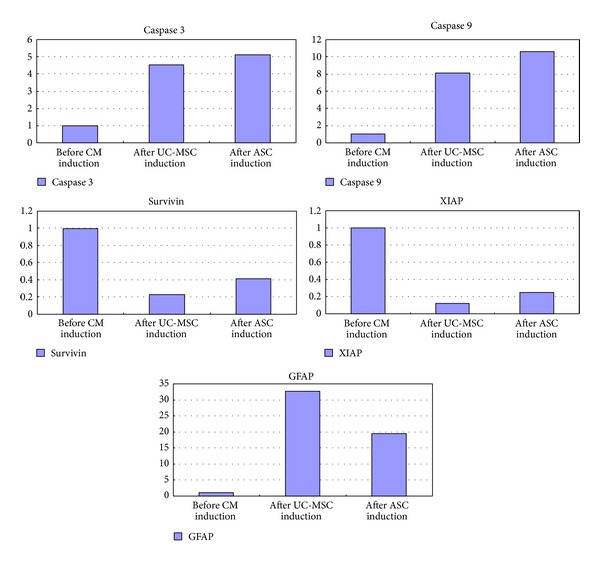
Quantitative real-time PCR of caspase 3, caspase 9, survivin, XIAP, and GFAP after MSCs conditioned medium induction. MSCs conditioned medium treatment leads to significant overexpression of caspase 3 and caspase 9 and significant downregulation of survivin and XIAP. GFAP expression is also significantly increased after induction.

XIAP (X-linked inhibitor of apoptosis protein) is usually overexpressed in U251 cells. After coculture, XIAP expression is greatly decreased, as is shown in Figure 4.

### 3.6. MSCs Conditioned Medium Coculture Induced Cell Cycle Arrest in U251 Cells

Cell cycle deregulation and apoptosis are closely related events, and disruption of cell cycle progression may ultimately lead to apoptotic death. The effects of MSCs conditioned medium on U251 proliferation were determined using cell cycle assay.

**Figure 5:**
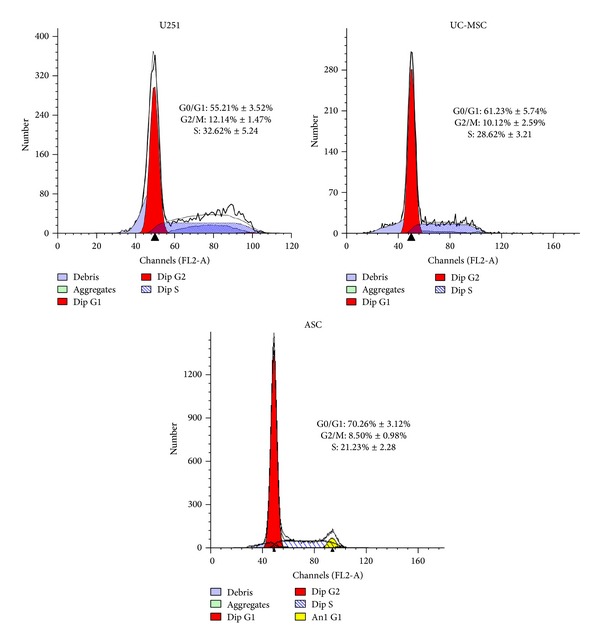
Cell cycle analysis of U251 cells after ASCs and UC-MSCs conditioned medium induction. MSCs conditioned medium treatment led to G0/G1 growth arrest in U251 cells.

MSCs conditioned medium increased the fraction of G0/G1 from 55.21 ± 3.52% in control to 61.23 ± 5.74% (for UC-MSC) and 70.26 ± 3.12% (for ASC) at the 48-hour time points (Figure 5). The G2-M and S phases were slightly decreased after treatment, indicating that MSC conditioned medium may induce G0/G1 growth arrest in U251 cells.

### 3.7. MSCs Conditioned Medium Coculture Leads to Significant Differentiation of U251 Cells towards a Normal Glial Cell Phenotype and Decrease in Tumor Infiltration Capability

**Figure 6:**
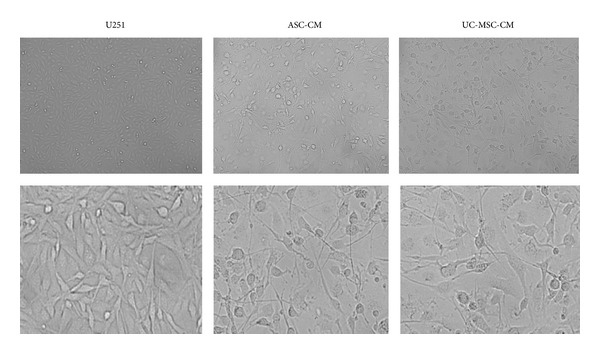
MSCs conditioned media coculture leads to significant differentiation of U251 cells towards a normal glial cell phenotype. Phase contrast image of U251 cells before and after MSCs conditioned media coculture. U251 glioma cells are usually with short processes. Much more and longer processes were seen after MSCs conditioned media induction and these processes connected with neighboring cells, forming an interconnected network.

Rather unexpectedly, MSCs conditioned medium also led to significant differentiation of U251 cells towards a normal glial cell phenotype. As early as 8 hours after conditioned medium treatment, U251 glioma cells experienced significant morphological change. U251 glioma cells usually have short processes. Many more processes were seen after MSCs conditioned medium induction and these processes interconnected with neighboring cells, as is shown in Figure 6. GFAP staining was performed on U251 cells before and after MSC medium exposure. Although U251 is a human glioma cell line, control U251 cells expressed very weak GFAP. However, after two days of growth in MSCs conditioned medium, GFAP expression became much more prominent and the outgrowing processes can be clearly stained, as is shown in Figure 8. These morphological changes led to a relatively normal glial cell phenotype. Quantitative real-time PCR for GFAP expression was performed. MSC conditioned medium treatment led to significant upregulation of GFAP mRNA, which is more than 35-fold, as is shown in Figure 4.

We are interested to see whether this relative normal morphology change also leads to a decreased tumor migration and infiltration capability.

**Figure 7:**
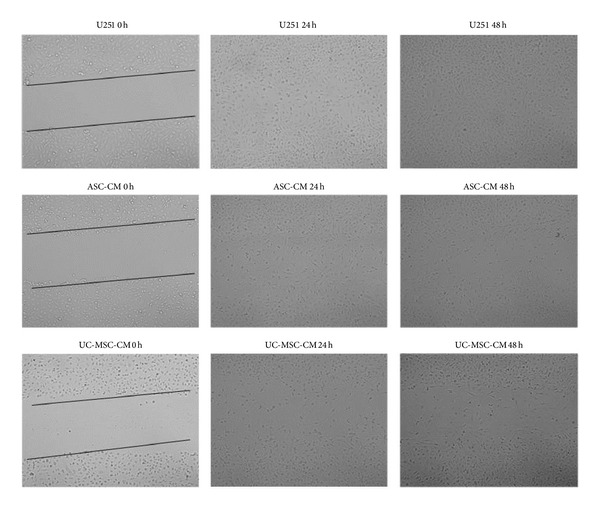
Scratch assay. U251 cells were induced with ASC-CM and UC-MSC-CM for two days. The cells quickly grew over the scratch for U251 cells cultured alone. After 48 hours, the scratch was invisible. For ASC-CM and UC-MSC-CM culture, although there are still some cells growing over the scratch line, the scratch is still visible. Fewer cells are seen grown over the line.

For tumor migration assay, a scratch assay was performed using U251 cells. After scratching, U251 cells soon grow over the scratch line, as if the scratch line never existed. But in the MSC conditioned medium group, only a few cells grew over the line, as is shown in Figure 7.

**Figure 8:**
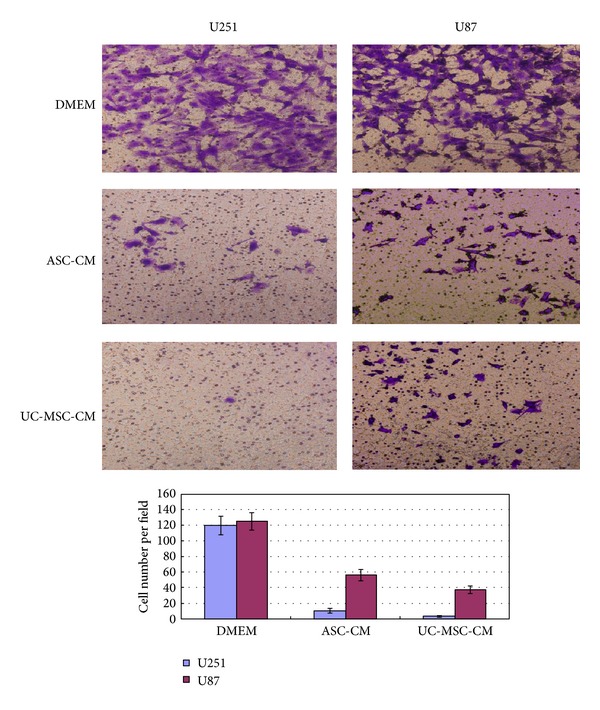
Matrigel invasion assay. U251 cells or U87 cells (induced or noninduced) were placed in transwells with 8 *μ*m pore size polycarbonate filters, precoated with Matrigel. The lower wells were filled with DMEM with 10% fetal bovine serum. The cells were incubated at 37°C for 24 hours. Nonmigratory cells on the upper surface of the transwells were removed and the migratory cells from the lower surface of the transwell were fixed and stained with 1% crystal violet. Cells were photographed and average cells per field were calculated. Experiments were done in triplicates on three times independently.

Next we used in vitro Matrigel invasion assay to determine whether the increased migration corresponded to an increase in invasive properties. We did this side by side with U87 cell line, another commonly used human glioma cell line. Noninduced control U251 and U87 cells can infiltrate the Matrigel. But after 2 days of conditioned media induction, only very few U251 cells can migrate through Matrigel, as is shown in Figure 8. There was a significantly decreased cell invasion capability of U251 cells after conditioned media culture and this difference is statistically significant. U87 cells also displayed decreased tumor infiltration capability but to a lesser extent.

**Figure 9:**
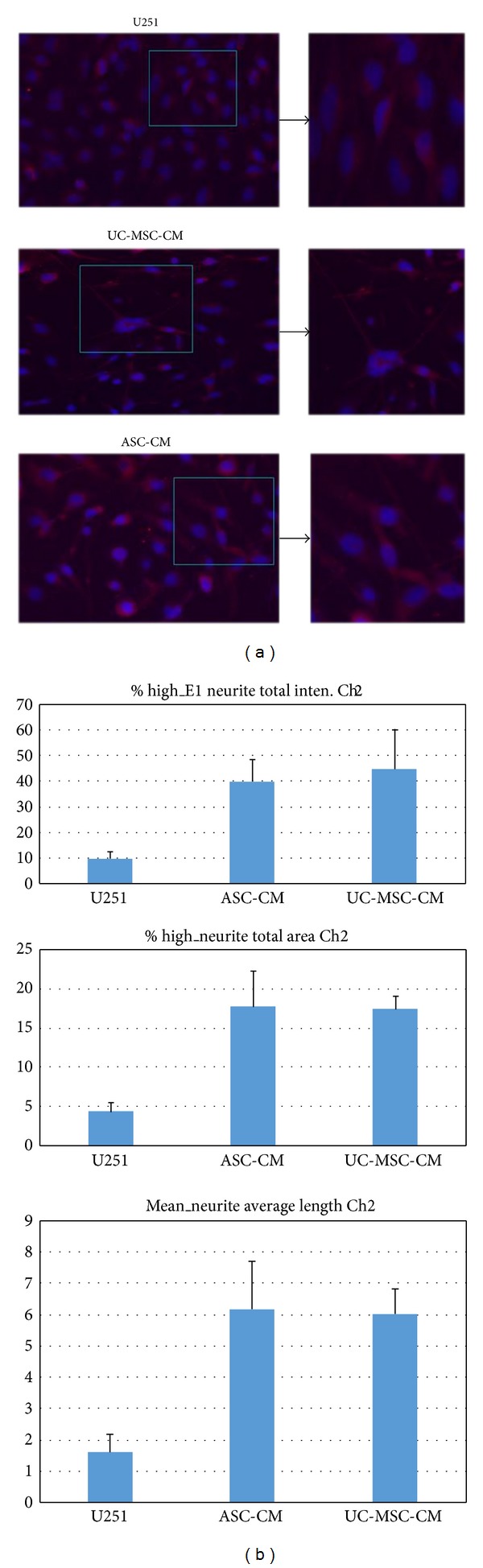
Cellomics high content screening (HCS) system analysis indicated much more prominent cell process outgrowth after MSCs conditioned media induction. (a) Representative fluorescent picture of U251 glioma cells before and after conditioned media induction. GFAP positive cells are shown in red. Much longer cell processes were seen after conditioned media induction. (b) Statistical analysis of cell process outgrowth before and after MSCs conditioned media induction. Both ASC- and UC-MSC-conditioned media led to more significant outgrowths of cell processes.

Cellomics high content screening (HCS) system analyses were performed to analyze the change in length of cell processes. Statistical analysis showed much more prominent cell processes outgrowth after MSCs conditioned media induction in U251 cells, as is shown in Figure 9.

### 3.8. Apoptotic Protein Microarray Analysis of ASC-CM and UC-MSC-CM

To find out what apoptotic related proteins are responsible for the increased apoptosis in U 251 cells, a human apoptosis antibody array serial analysis were carried out. Lysates of U251 cells before and after MSC conditioned medium induction were examined by high-throughput protein array in which 43 human apoptosis related proteins were examined in one array. Representative Apoptotic Protein microarray analyses are shown in Table 2. As expected, UC-MSCs and ASCs conditioned medium induction led to significant overexpression of pro-apoptotic proteins such as Bad, Bax, Bim, Bid, etc. and significant downregulation of anti-apoptotic proteins such as Bcl-2, Survivin and XIAP.

## 4. Discussion

Human malignant gliomas are the most commonly diagnosed malignant adult primary brain tumors. Resistance to induction of cell death by apoptosis in response to radiotherapy or chemotherapy is one of its cardinal characteristics [20]. Most glioma patients have very unfavorable expectation upon diagnosis, whereas for GBM (glioblastoma multiforme) patients, a period of only 3–6 months is expected. With such a miserable prognosis, searching for new treatment is necessary.

Mesenchymal stem cells (MSCs) are a population of stem cells with great self-renewal and multipotentiality. MSCs are potential seed cells in regenerative medicine and have been used for treatment of various neurological diseases such as spinal cord injury and cerebral palsy [21]. Current research for exploration of MSCs in cancer therapy mainly uses MSCs as a vehicle for targeting tumor cells, because these cells have been found to have tumor chemotactic capabilities and can migrate towards tumor sites [22–24]. When transduced with therapeutic molecules such as IFN-a, IFN-b, TRAIL, and thymidine kinase (TK), anticancer activity was often observed in MSCs [25–27]. But few studies investigated MSCs without transducing these targeting molecules. Akimoto et al. reported U251 growth inhibition when cocultured with UC-MSCs but growth promotion when cocultured with ASCs [28]. Recently, Behnan et al. reported that MSCs might contribute to glioma tumor progression [29]. These contradictory results might be due to different cell sources being used (primary tumor cells or tumor cell line), different culture methods, different experimental condition and designs, and so forth. Given the fact that MSCs have been widely used in various studies as tumor-targeting vehicles with obvious effects, it would be problematic if MSCs themselves are promoting tumor growth. Therefore we reasoned MSCs themselves might have some antitumor properties. In our preliminary experiments using transwell coculture system, we did see growth inhibition of U251 human glioma cells when cocultured with MSCs. The same effect can be observed simply by using conditioned media coculture from MSCs. Therefore we designed this ex vivo study. For MSCs, we compared two easily accessible MSCs sources, namely, human adipose tissue-derived mesenchymal stem cells (ASCs) and umbilical cord-derived mesenchymal stem cells (UC-MSCs), in parallel. We wanted to see whether different sources of MSCs have impact on growth kinetics, apoptotic rate, pro- and antiapoptotic gene expressions, and so forth.

Our study showed conditioned media from both types of MSCs can significantly inhibit the growth of human U251 cell line, especially for UC-MSCs, which inhibited more than 50% of growth of U251 cells. To find out whether this is due to growth retardation of U251 cells or increased apoptosis, we did annexin and PI flow cytometric assay. Flow cytometric assay indicated both types of MSCs can significantly induce the apoptosis in human U251 cell line, which is more true for UC-MSCs. The susceptibility of tumor cells to induction of apoptosis is usually controlled by the ratio between proapoptotic and antiapoptotic proteins. Induction of apoptosis usually requires involvement of the bcl-2 family proteins including proapoptotic gene products (e.g., bad, bax, bak, bcl-XS, and bim) and apoptosis inhibiting gene products (e.g., bcl-2, bcl-XL) [30, 31]. On the other hand, caspases, especially caspase 3, play a key role during the execution phase in various forms of apoptosis [32].

To get a comprehensive picture of the apoptotic related factors during induction of apoptosis in U251 cell lines, we did RayBio Biotin Label-Based Human Apoptosis Array I (Cat number AAH-APO-G1-4: Norcross, GA), which can detect the expression levels of 43 human apoptotic related proteins in cell culture supernatants simultaneously. These apoptotic related proteins include bcl-2, bax, bad, caspase 3, caspase 8, survivin, XIAP, p53, p21, Fas, TRAIL, and TNF-beta. As expected, both MSCs conditioned media secreted much more prominent apoptotic related proteins, especially for bad, bax, bak, and bim. Survivin and XIAP are significantly lower in MSCs conditioned medium culture as compared to U251 single culture alone. Notably we also saw significant overexpression of TNF receptor after MSC conditioned medium induction. It is plausible that, during the induction of apoptosis by MSCs, TNF signalling pathway also played an important role.

Next we did quantitative real-time PCR experiments. We found significant upregulation of apoptotic genes of both caspase 3 and caspase 9 and significant downregulation of antiapoptotic genes such as survivin and XIAP after MSC conditioned medium induction in human U251 cell line. This result is consistent with our protein array data.

The regulation of cell proliferation and terminal differentiation is a critical aspect of normal development and homeostasis but is frequently disturbed during tumorigenesis. Cell proliferation and differentiation are specifically controlled in the G1 phase and the G1/S phase transition in the cell cycle [33]. When we did cell cycle analysis on U251 cells prior to and after the induction, we found significantly more cells in G0/G1 phase after induction. Therefore, we have reason to believe MSC conditioned medium might repress cell growth via cell cycle arrest in the G0/G1 phase.

During the induction experiments, rather unexpectedly, we found MSC conditioned media induced rapid (as early as 6–8 hours after induction) and complete differentiation in U251 cells. This means MSCs conditioned medium triggers cell transformation, indicative of the cells' differentiation into a more mature astrocytic state. This differentiation potential is further confirmed by an increased expression of GFAP, a 50 kDa type III intermediate filament protein considered to be a reliable differentiation marker of normal astrocytes [34]. In glial tumors, GFAP expression is frequently lost with increasing grade of malignancy, suggesting that GFAP is important for maintaining glial cell morphology or regulating astrocytoma cell growth. Increased GFAP expression after MSC conditioned medium induction is evidenced by significant GFAP fluorescent staining and significant upregulation of GFAP genes using real-time PCR (more than 35-fold increase in expression). These results indicate human mesenchymal stem cells can efficiently induce both apoptosis and differentiation in U251 human glioma cell line. Whereas UC-MSCs are more efficient for apoptosis induction than ASCs, their capabilities of differentiation induction are not distinguishable from each other.

Differentiation therapy, using agents that modify cancer cell differentiation, has shown to be effective and became the standard therapy for acute promyelocytic leukemia (APL), which uses either all-*trans*-retinoic acid or the inorganic toxicant arsenic trioxide (As_2_O_3_), a well-known environmental carcinogen. Both drugs take effect by triggering apoptosis and differentiation of APL cells in a dose-dependent manner [35–38]. Such excellent effects, however, were not reproduced in other hematological and, particularly, solid tumors. Differentiation agents for malignant gliomas remain a real challenge. Some studies have shown arsenic trioxide (As_2_O_3_) and forskolin can also trigger apoptosis and differentiation in glioma cells [39, 40]. In our study, we unexpectedly found a very strong differentiation effect of conditioned MSCs medium. More impressively, the tumor infiltration capabilities of U251 cells were greatly reduced after induction, as is shown in Matrigel infiltration assay. The tumor infiltration capabilities of U87 cells were also reduced after induction but to a lesser extent. We are planning to do further experiments to look deeper into this phenomenon.

In this study, we showed conditioned media from human mesenchymal stem cells can efficiently induce apoptosis in U251 human glioma cell line. Furthermore, MSC conditioned medium is a powerful astrocytic differentiation-inducing agent in cultured human U251 glioma cell line. Our findings suggest MSCs themselves have favorable antitumor characteristics and should be further explored in future glioma therapy. Future studies, however, should extend these findings to in vivo tumor models.

## Supplementary Material

Morphology and phenotypic characterization of UC-MSCs and ASCs.

## Figures and Tables

**Table 1:** Primers used for real-time PCR.

	Primer sequences	
	Forward	Reverse
Caspase 3	CAGTGGAGGCCGACTTCTTG	TGGCACAAAGCGACTGGAT
Caspase 9	TGTCCTACTCTACTTTCCCAGGTTTT	GTGAGCCCACTGCTCAAAGAT
Survivin	ACCACCGCATCTCTAC	TCCTCTATGGGGTCGT
XIAP	GGCCAGACTATGCCCATTTA	CGAAGAAGCAGTTGGGAAA
Actin F	GTCGTACCACTGGCATTGT	CAGCTGTGGTGGTGAAGCT

**Table 2:** Representative RayBio apoptosis assay overview (in part).

Protein name	U251 alone	ASC CM	UC-MSC-CM	Fold change 1	Fold change 2
Bad	10.05	181.61	203.00	18.07	20.20
Bax	192.00	265.05	363.00	1.38	1.89
Bcl-2	430.00	206.15	175.48	0.48	0.41
Bcl-w	350.00	310.80	286.03	0.89	0.82
BID	185.00	208.00	281.10	1.12	1.52
BIM	231.00	240.51	387.15	1.04	1.68
Caspase 3	235.00	932.60	881.00	3.97	3.75
Caspase 8	200.00	392.67	677.00	1.96	3.39
Fas	1271.50	1638.43	2100.05	1.29	1.65
FasL	274.50	269.46	177.42	0.98	0.65
p21	179.00	208.61	183.37	1.17	1.02
p27	240.00	333.80	238.20	1.39	0.99
p53	255.00	319.05	252.66	1.25	0.99
SMAC	407.50	1548.60	1535.00	3.80	3.77
Survivin	205.00	114.50	124.00	0.56	0.60
sTNF-R1	95.00	272.91	413.53	2.87	4.35
sTNF-R2	132.00	138.07	204.46	1.05	1.55
TNF-alpha	287.00	294.50	280.37	1.03	0.98
TNF-beta	246.00	349.48	306.32	1.42	1.25
XIAP	200.74	101.02	125.95	0.50	0.63

These readouts are the readouts after subtracting the background. Signal strength fold change larger than 1.5 or smaller than 0.7 is considered statistically significant. UC-MSCs and ASCs conditioned media led to significant overexpression of proapoptotic proteins such as bad, bax, BIM, and BID and significant downregulation of antiapoptotic proteins such as bcl-2, survivin, and XIAP.
