# Supplementary material for: Conditioned Media from Human Adipose Tissue-Derived Mesenchymal Stem Cells and Umbilical Cord-Derived Mesenchymal Stem Cells Efficiently Induced the Apoptosis and Differentiation in Human Glioma Cell Lines In Vitro
Source: Biomed Res Int. 2014 May 27;2014:109389. doi: 10.1155/2014/109389 (PMC4058294; doi:10.1155/2014/109389)
Supplement: Supplementary file 1 — Morphology and phenotypic characterization of UC-MSCs and ASCs. [file 109389.f1.pdf]

Supplementary data:

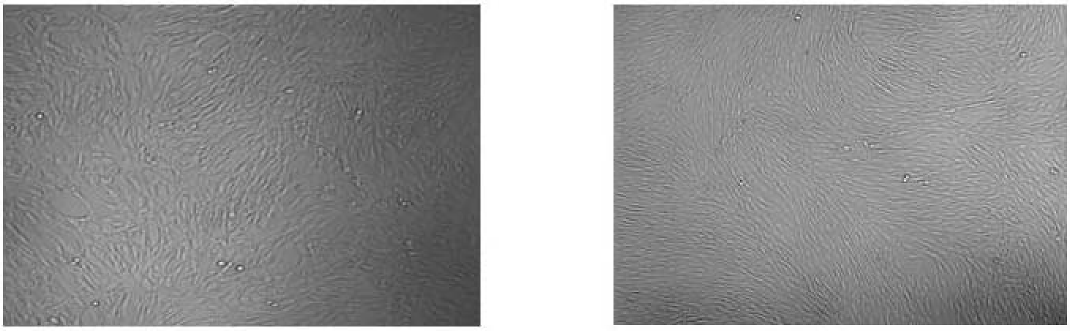

UC-MSC ASC

Supplementary Figure 1. Morphology of UC-MSCs and ASCs ex vivo at Passage 3. UC-MSC: human umbilical cord derived mesenchymal stem cell; ASCs: adipose tissue derived mesenchymal stem cell. Both type of cells displayed a fibroblast-like morphology.

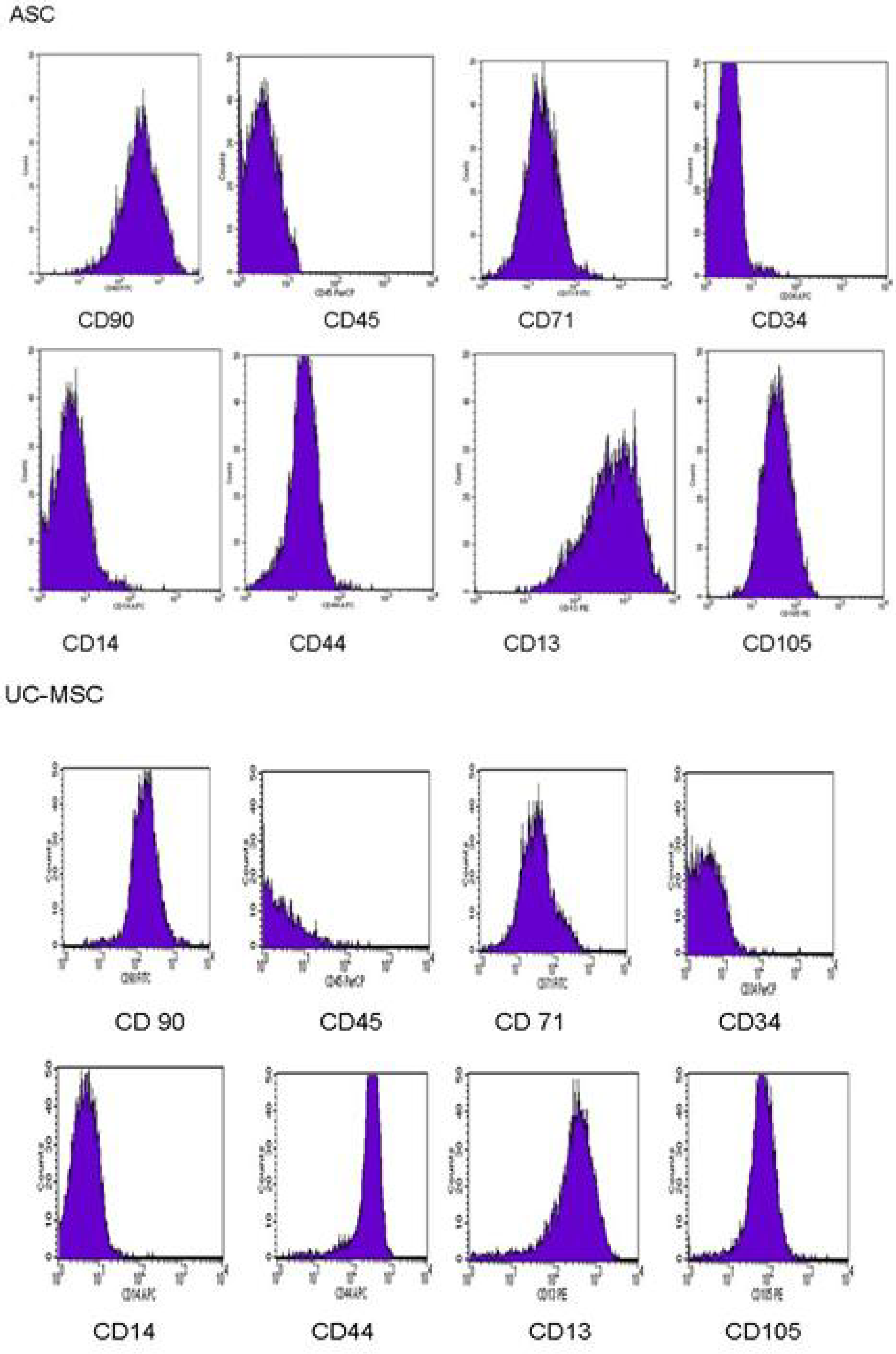

Supplementary Figure 2. Immunophenotyping of ASCs and UC-MSCs. ASCs and UC-MSCs both exhibited positive surface antigenicity for CD13, CD44, CD71, CD90, CD105, and exhibited negative surface antigenicity for CD14, CD45 and CD34.

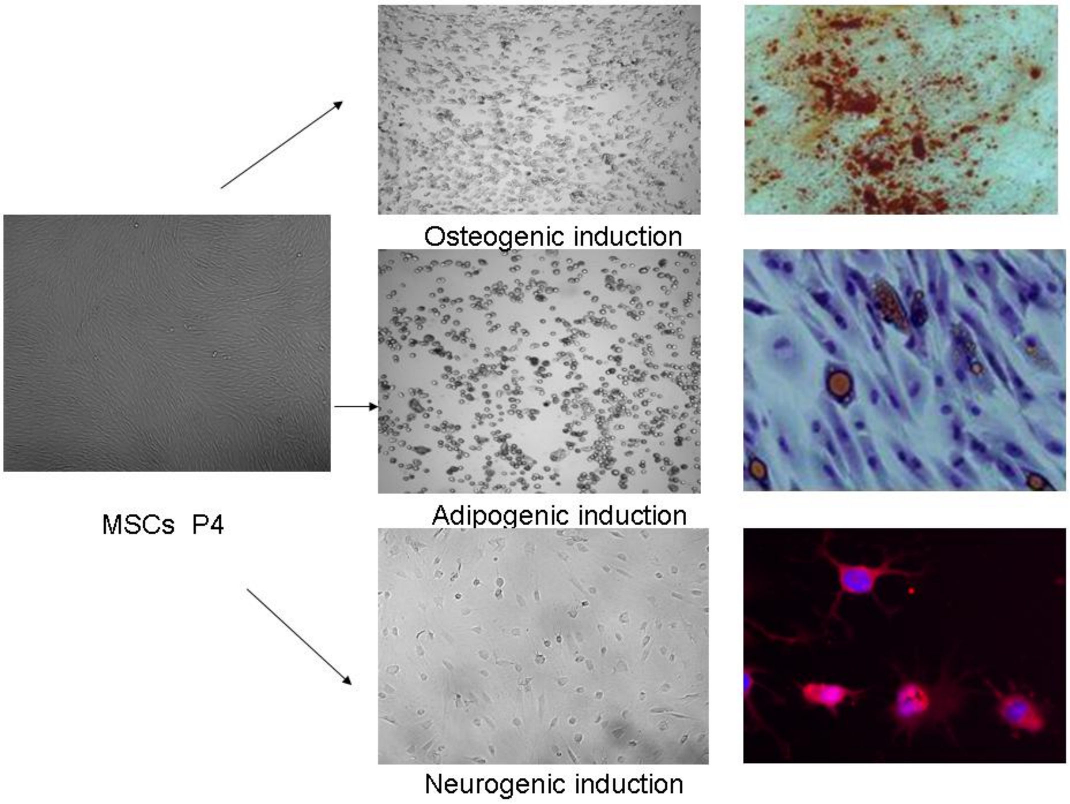

Supplementary Figure 3. Multi-lineage Differentiation of Mesenchymal Stem Cells (MSCs). ASCs and UC-MSCs were induced to differentiate into osteocytes, adipocytes and neurons, as is shown in light microscopy and in Alizarin red staining, Oil-Red-O staining and immunofluorescent staining (NSE is shown in red fluorescence).
